# Burden and Clinical Impact of Hepatitis D Virus Co-Infection Among HBsAg-Positive Patients in Mauritania

**DOI:** 10.3390/diseases14020069

**Published:** 2026-02-12

**Authors:** Mohamed Abdawa, Mohamed Hemeyine, Isabelle Chemin, Françoise Lunel-Fabiani, Mohamed Vall Mohamed Abdellahi

**Affiliations:** 1 Unité d’Épidémiologie Moléculaire et Diversité des Micro-Organismes (EMDM), Université de Nouakchott, Nouakchott, Mauritania; 2 Institut National d’Hépato-Virologie (INHV), Nouakchott, Mauritania; 3 Institut National de la Santé et de la Recherche Médicale (INSERM) U1350, 151 Cours Albert Thomas, 69003 Lyon, France; 4 HIFIH UFR Santé, Faculté de Médecine, University of Angers, 4 Rue de Larrey, 49933 Angers, France

**Keywords:** hepatitis B, hepatitis D virus, cirrhosis, hepatocellular carcinoma, Mauritania, Africa

## Abstract

Background: Hepatitis B virus (HBV) infection remains highly endemic in sub-Saharan Africa, where hepatitis delta virus (HDV) co-infection substantially worsens liver disease outcomes. Mauritania has long been suspected to be a high-burden setting for HBV-HDV co-infection, yet contemporary data describing its clinical and virological impact remain limited. Methods: We conducted a hospital-based cross-sectional study at the National Institute of Hepato-Virology (INHV) in Nouakchott, including 401 HBsAg-positive patients. Demographic, clinical, biological, and virological data were collected. HDV serology and RNA testing were performed when available. Liver disease severity, including cirrhosis and hepatocellular carcinoma (HCC), was assessed using clinical, biological, and imaging criteria. Results: HDV antibodies were detected in 31.9% of HBsAg-positive patients, confirming Mauritania as a hyper-endemic area for HDV. HDV co-infection was strongly associated with advanced liver disease, with HDV antibodies present in 86.4% of cirrhotic patients and 82.4% of those with HCC. Patients with HDV infection frequently exhibited suppressed HBV DNA levels, reflecting viral interference. A substantial proportion of patients presented with decompensated cirrhosis or HCC at diagnosis, and nearly 70% were treatment-naïve. Overall, HDV co-infection emerged as the principal driver of severe liver disease in this cohort. Conclusions: HBV/HDV co-infection is highly prevalent in Mauritania and is associated with a wide clinical spectrum ranging from asymptomatic infection to decompensated cirrhosis and hepatocellular carcinoma. HDV co-infection is the principal driver of severe liver disease, often occurring despite low or undetectable HBV DNA levels. Systematic HDV screening among all HBsAg-positive individuals is urgently needed to improve risk stratification, guide therapeutic decisions, and reduce liver-related morbidity and mortality.

## 1. Introduction

Hepatitis B virus (HBV) infection remains a major global health problem, with an estimated 296 million people living with chronic infection in 2019 and approximately 820,000 deaths each year, mainly due to cirrhosis and hepatocellular carcinoma (HCC) [1,2]. The burden is particularly high in sub-Saharan Africa, where early-life transmission sustains HBsAg prevalence rates frequently exceeding 10% [3,4]. Mauritania, a West African country with high HBV endemicity, exemplifies this challenge, with reported HBsAg seroprevalence ranging from 10.7% among pregnant women to 18.3% in selected patient populations [5].

In this setting, hepatitis delta virus (HDV) co-infection represents a critical but underrecognized determinant of disease severity. HDV affects up to 20% of HBsAg-positive individuals in parts of sub-Saharan Africa and is associated with accelerated liver fibrosis, early cirrhosis, and a markedly increased risk of HCC compared with HBV mono-infection [5,6,7]. In Mauritania, although active HBV/HDV co-infection has been reported in approximately 3.8% of HBsAg carriers, historical exposure to HDV, reflected by anti-HDV seropositivity, has been strongly linked to severe fibrosis and a threefold increase in HCC risk [2,8,9].

Clinically, chronic HBV and HBV/HDV co-infection present with a wide spectrum of manifestations, ranging from asymptomatic disease and mild biochemical abnormalities to advanced cirrhosis and HCC. Disease progression is influenced by demographic factors, viral replication profiles, co-infections, and host-related determinants, with cirrhosis prevalence reported between 4% and 13% regionally and annual HCC incidence reaching 2–5% among cirrhotic patients [4,10,11]. Virologically, outcomes are shaped by HBeAg status, HBV DNA levels, viral genotypes—predominantly genotype E in West Africa—and the presence of HDV, which profoundly alters the natural history of HBV infection [5,6,7].

Despite the recognized severity of HBV/HDV co-infection, comprehensive data integrating clinical presentation and virological markers among HBsAg-positive individuals in Mauritania remain limited. In particular, the interplay between HDV infection, HBV replication, and advanced liver disease has not been fully characterized at the population level [12,13].

Therefore, this study aimed to describe the clinical and virological characteristics of HBsAg-positive patients in Mauritania, with a specific focus on HBV and HDV co-infection. By analyzing demographic features, disease stage, liver function parameters, and viral markers, this work seeks to inform targeted screening strategies, optimize clinical management, and support future prevention and treatment efforts in this high-burden setting [5,8,14].

## 2. Materials and Methods

### 2.1. Samples and Methods

#### 2.1.1. Study Population

This study included a total of 401 patients who tested positive for hepatitis B surface antigen (HBsAg) and were by chance seen in consultation at National Hepato-Virology Institute (INHV), Nouakchott, between July 2023 and December 2025. All participants voluntarily provided blood samples after giving informed consent. Epidemiological, demographic, and clinical information was collected using a standardized clinical data collection form.

The study protocol was reviewed and approved by the Ethics Committee of the Ministry of Health (approval reference 012-2025, granted on 7 July 2025) after completion of the administrative processing of an application submitted before the start of participant enrolment. Written informed consent was obtained from all participants prior to enrollment, in accordance with national ethical guidelines and the principles of the Declaration of Helsinki. Laboratory results, including HDV status, were systematically communicated to both patients and their treating physicians to support routine clinical management in accordance with local practice and international recommendations.

#### 2.1.2. Data Collection and Clinical Assessment

Demographic and clinical data were recorded for each patient, including age, sex, ethnic group, marital status, lifestyle characteristics, occupation, and presenting clinical features. Diagnoses of liver cirrhosis and hepatocellular carcinoma (HCC) were established and validated by attending physicians at INHV, based on clinical evaluation, imaging, and laboratory findings.

Medical information integrated into the epidemiological record included abdominal ultrasonography results, documented clinical signs and biochemical and hematological parameters. These included serum transaminases (ALT and AST), total and direct bilirubin, urea and creatinine levels, and alpha-fetoprotein concentration. Antiviral treatment decisions were made by treating physicians according to local clinical practice and drug availability, with tenofovir primarily prescribed for HBV-related indications (including cirrhosis or HBV DNA > 2000 IU/mL), and pegylated interferon-α used for HDV infection when available.

#### 2.1.3. Detection of HBsAg and Anti-HDV Antibodies

Plasma samples (500–1000 µL) were collected from all patients and stored at −20 °C until analysis. HBsAg detection was performed using the VIDAS HBs Ag Ultra assay on the mini VIDAS platform (bioMérieux, Marcy l’Étoile, France). Total antibodies against hepatitis D virus (anti-HDV) were detected using a commercial ELISA kit (Eurofins Biomnis, Ivry-sur-Seine, France).

#### 2.1.4. Quantification of HBV and HDV Viral Loads

HBV DNA was extracted from plasma samples using the QIAGEN EZ1 Advanced XL automated extraction system, based on magnetic-particle technology, following the manufacturer’s recommendations. Nucleic acids were purified using the EZ1 Virus Mini Kit v2.0 and eluted in the appropriate elution volume for downstream molecular analysis. Quantification of HBV viral load was performed by real-time PCR (qPCR) using the Rotor-Gene Q platform (QIAGEN, Hilden, Germany). Amplification and detection were carried out with a commercial HBV quantitative PCR kit including an internal control and calibration standards, and results were expressed as IU/mL according to the kit instructions. The lower limit of detection (LOD) for HBV DNA was 3.8 IU/mL. Positive and negative controls were included in each run to ensure assay validity and to monitor potential contamination.

Hepatitis D virus (HDV) RNA was assessed in plasma samples from patients who tested positive for hepatitis D antibodies (HDAb) using a commercial real-time reverse transcription PCR (RT-PCR) assay (EurobioPlex^®^ Hepatitis Delta, Eurobio Scientific, Les Ulis, France). Viral load results were expressed in IU/mL and log IU/mL. According to the manufacturer’s specifications, the lower limit of detection of the assay is 150 IU/mL (approximately 2.2 log IU/mL). Quality control procedures were performed in accordance with the laboratory’s accreditation requirements.

### 2.2. Statistical Analysis

All statistical analyses were carried out using JASP (version 0.19.3). Quantitative variables are summarized using either means with standard deviations or medians with interquartile ranges, depending on data distribution. Comparisons between groups were conducted using parametric or non-parametric tests as appropriate, including Student’s *t*-test, Welch’s *t*-test, and the Mann–Whitney *U* test. Relationships between categorical variables were evaluated using the chi-square test or Fisher’s exact test when applicable. Statistical significance was defined as a two-sided *p* value below 0.05. Graphs and figures were generated using R statistical software (version 4.5.2; R Foundation for Statistical Computing, Vienna, Austria), with visualizations produced using the ggplot2 package (version 4.0.1).

### 2.3. Sample Size Estimation

The minimum required sample size was calculated using the Schwartz formula for prevalence studies:

$$n\;=\;\frac{Z^{2}P(1-P)}{d^{2}}$$

For liver cirrhosis, a prevalence of 10% among HBsAg-positive individuals in Mauritania was assumed, based on data from sub-Saharan Africa [3,5] and the high burden of HDV co-infection in the country [4]. Using a 95% confidence level and a precision of 3%, the minimum sample size was estimated at 385 carriers.

For hepatocellular carcinoma, prevalence estimates in the same population ranged from 2%, corresponding to a minimum sample size of 257 carriers under similar assumptions, to a more conservative estimate of 5% [4,8], yielding a required sample size of 203 carriers. Considering these estimates and the likelihood of underestimation of HCC prevalence in resource-limited settings, the larger sample size of 385 participants was retained to ensure adequate representation of both cirrhosis and hepatocellular carcinoma.

### 2.4. Use of AI Tools

AI tools were used solely for translation and to improve the clarity of the manuscript’s language. All content was reviewed and verified by the authors, who take full responsibility for the integrity and accuracy of the work. No AI tools were used for study design, data collection, analysis, interpretation, or the drawing of conclusions.

## 3. Results

### 3.1. Demographic Characteristics and Clinical Spectrum

As shown in Table 1, a total of 401 HBsAg-positive patients were included. The mean age of the cohort was 40.5 ± 12.1 years, and most patients were young to middle-aged adults: 44% were between 25–40 years and 43% were between 40–65 years. Adolescents and young adults (17–25 years) accounted for roughly 10%, whereas only 3% of participants were over 65 years of age. The sex distribution was nearly balanced (50.6% men vs. 49.4% women). The population was predominantly White Moor (69.3%), with Black Moor patients making up 30.7% of the cohort. About 38% of patients lived in urban settings and 32% in rural settings; the remainder had missing data. Most participants were married (77.3%), and just over half were formally educated (51.6%). Employment in the trade/private sector was the most frequent occupation (23.9%).

The majority of patients (66.1%, 95% CI 61.2–70.7) had chronic hepatitis B (HBsAg positivity persisting for ≥6 months), while 27.4% (95% CI 23.1–32.1) had progressed to liver cirrhosis and 4.2% (95% CI 2.5–6.7) had hepatocellular carcinoma (HCC) (Table 2).

### 3.2. Clinical Characteristics of the Cohort

Liver function tests were moderately elevated. The median aspartate aminotransferase (AST) was 34 U/L (IQR = 66), and the median alanine aminotransferase (ALT) was 38 U/L (IQR 53.8). Median total bilirubin was 12.05 mg/L (IQR 12.05), and the prothrombin time was 86% (IQR 22). Gamma-glutamyl transferase (γ-GT) levels had a median of 62 U/L (IQR 60.5), while the alpha-fetoprotein (AFP) median was 10.1 ng/mL (IQR 18.45). Renal function indices were largely within normal limits (median urea 0.32 g/L, IQR 0.195; median creatinine 11 mg/L, IQR 4.7).

About 19.9% of patients presented with signs of liver decompensation such as ascites or encephalopathy. Another 11.5% had jaundice, while the majority (68.6%) were asymptomatic at the time of evaluation. Abdominal ultrasonography revealed abnormal findings in approximately 17% of patients, and an additional 26% showed signs of portal hypertension, such as splenomegaly or varices. However, more than half (56.9%) had a normal ultrasound. Most patients (69.6%) were treatment-naïve at the time of assessment. Among treated individuals, 25.2% were receiving pegylated interferon-α (PEG-INFα) monotherapy, 3.2% received PEG-INFα combined with tenofovir disoproxil fumarate (TDF), and 2% received TDF alone. The high proportion of untreated patients indicates limited access to or uptake of antiviral therapy despite the high prevalence of active or advanced disease (Table 3).

**Figure 1:**
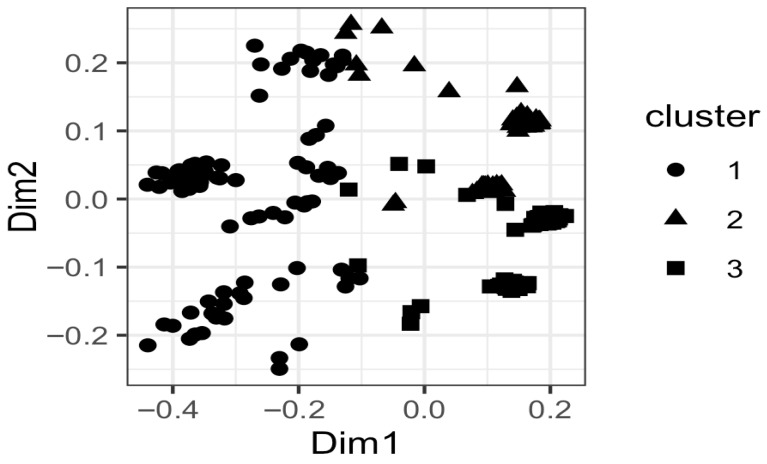
Patient clustering based on clinical, biochemical and virological characteristics. Clusters were derived using partitioning around medoids (PAM) on Gower distance and visualized using principal coordinate analysis. Dim 1 and Dim 2 represent the first two principal coordinates, accounting for the largest proportion of variability in the dataset. Each point represents one patient, and symbols indicate cluster membership.

To further explore patterns of clinical, biochemical and virological characteristics across the cohort, an unsupervised clustering analysis was performed based on demographic, clinical, biochemical and virological data, resulting in the identification of three distinct patient clusters. Cluster 1 (*n* = 91) comprised exclusively patients with cirrhosis or hepatocellular carcinoma and was predominantly HDAb-positive, with a higher proportion of undetectable HBV-DNA and nearly all patients receiving antiviral treatment. Cluster 2 (*n* = 75) consisted mainly of patients with chronic hepatitis B without cirrhosis or HCC, showed intermediate biochemical profiles and mixed HBV-DNA detectability, and was largely treatment-naïve. Cluster 3 (*n* = 106) included predominantly younger patients with chronic hepatitis B, more frequent detectable HBV-DNA, and minimal HDV involvement and was mostly treatment-naïve (Figure 1).

### 3.3. HDV Sero-Prevalence

In the total cohort, 31.9% tested positive for total hepatitis D antibodies (HDAb), and 27.7% were HDAb-negative. Among the 239 patients with available HDAb status, 128 were identified as HDAb-positive and 111 as HDAb-negative, indicating a seroprevalence of hepatitis delta of 53.6% (Table 3). Those who tested positive for HDAb were, on average, older (43.5 ± 11.4 years vs. 40.1 ± 11.5 years, *p* = 0.025) and exhibited a higher proportion of males. White Moors constituted the predominant demographic (approximately 80%) of HDAb-positive patients, whereas HDAb-negative individuals were more frequently Black Moors (56%).

**Figure 2:**
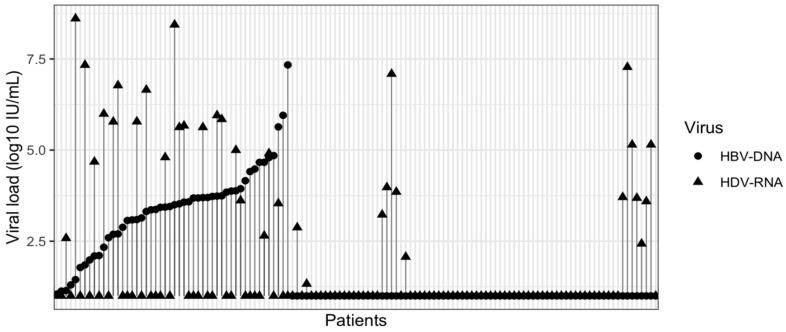
Distribution of HBV-DNA and HDV-RNA viral loads among HDAb-positive patients.

Clinically, HDAb-positive patients were more likely to have signs of liver decompensation and jaundice and were less likely to be asymptomatic compared with HDAb-negative patients. Biochemical markers were markedly worse in the HDAb-positive group: AST, ALT, γ-GT, AFP, bilirubin and prothrombin time were all significantly higher (*p* < 0.001), consistent with more severe liver disease. HBV DNA was more often undetectable in HDAb-positive patients, whereas HDAb-negative patients had higher rates of detectable HBV DNA. Among HDAb-positive patients, HDV-RNA levels were highly heterogeneous and often elevated despite low or undetectable HBV-DNA, indicating active HDV replication independent of HBV viral load (Figure 2). This virological pattern is consistent with immune-mediated suppression of HBV replication in the presence of HDV. Accordingly, HDAb-positive individuals were significantly more likely to have cirrhosis or HCC and to be receiving antiviral treatment, whereas HDAb-negative patients were more frequently treatment-naïve (Table 4).

A multivariable logistic regression analysis was conducted among patients with available HDV serology (*n* = 239) to identify factors independently associated with advanced liver disease (cirrhosis or hepatocellular carcinoma). Results of the adjusted model are shown in Table 5. HDV antibody positivity was associated with substantially increased odds of cirrhosis/HCC compared with HDV-negative status (aOR 18.4), although the association did not reach conventional statistical significance (95% CI 0.96–351.3; *p* = 0.053).

### 3.4. HDV and HBV Quantification

Among patients with HDV RNA testing, half (50.8%) had detectable HDV RNA with a median viral load of 270 IU/mL (IQR 140,390), indicating detectable HDV replication in 50.8% of HDAb-positive patients, whereas the remainder had undetectable HDV RNA (Table 3).

HBV DNA was detectable in 58.1% of patients, with a median viral load of 876 IU/mL (IQR 4372.5). Notably, 22.4% of the cohort (*n* = 90) had HBV DNA levels > 2000 IU/mL (median 5723 IU/mL, IQR 42,739), which is a recognized threshold for initiating antiviral therapy. Conversely, 41.9% had undetectable HBV DNA (Table 3).

### 3.5. Prognosis of Cirrhosis and HCC Patients

The prognosis of patients with cirrhosis and hepatocellular carcinoma (HCC) was evaluated based on their demographic, clinical, virological, and biological profiles (Table 6). Among the 127 patients with advanced liver disease, 110 had cirrhosis and 17 had HCC. HCC patients were roughly a decade older than those with cirrhosis (mean age 52.2 ± 8.9 years vs. 42.7 ± 10.8 years), with a male predominance in both groups (66.4% and 76.5%, respectively). Most patients belonged to the White Moor ethnic group and lived in urban areas, and the majority were married. Clinically, signs of liver decompensation were present in 61.8% of cirrhotic patients and 70.6% of HCC patients, while jaundice was observed in 20.9% and 11.8%, respectively, and approximately 17% of patients in both groups were asymptomatic. Biological assessment showed elevated median transaminase levels (AST and ALT), increased γGT levels, impaired coagulation reflected by reduced prothrombin time, and elevated bilirubin levels. AFP concentrations were markedly increased in patients with HCC. Renal function parameters, including urea and creatinine, were mildly elevated. A high prevalence of hepatitis delta antibody positivity was observed in both cirrhosis (86.4%) and HCC (82.4%), with detectable HDV RNA in 39.1% and 35.3% of cases, respectively. HBV DNA was detectable in the majority of patients, including viral loads above 2000 IU/mL. Most patients had received antiviral treatment, predominantly pegylated interferon-α, either alone or in combination with tenofovir, while a small proportion were treated with tenofovir alone or remained treatment-naïve. Overall, these findings indicate an advanced stage of liver disease with significant virological activity, reflecting a poor prognosis, particularly among patients with HCC.

## 4. Discussion

Our study, conducting a comprehensive clinical and virological characterization of 401 HBsAg-positive patients in Nouakchott, confirms that Mauritania remains a hyper-endemic zone for both hepatitis B and hepatitis delta viruses [5]. The findings reveal a distinct epidemiological landscape characterized by a high prevalence of HDV co-infection (31.9%), which acts as the primary driver of severe liver disease—specifically cirrhosis and hepatocellular carcinoma (HCC)—in this population. Consistent with this, nearly half of the cohort already had cirrhosis and HCC was present in a substantial proportion, approaching rates reported in other high-burden African and North African settings [13,15,16].

The overall HDV seroprevalence of 31.9% observed in our cohort is strikingly high compared to global estimates, which typically range from 4.5% to 13% among HBsAg carriers [17]. Even within the West African context, Mauritania appears to represent a unique “hotspot” of high endemicity. While neighboring countries like Senegal, The Gambia, and Burkina Faso report HDV prevalence rates between 2% and 10% in general HBsAg-positive populations [18,19], our data align more closely with the historical “Mauritanian profile” described in smaller cohorts [8] and high-burden regions in Central Africa [17]. The high proportion of patients with detectable HDV RNA further supports ongoing transmission and the pathogenic role of active HDV replication in this setting [5]. This distinct geographical clustering suggests that specific local transmission dynamics, potentially intrafamilial or iatrogenic, maintain this high reservoir of infection.

A crucial finding of our study is the strong virological interference observed between HDV and HBV. We found that HDAb-positive patients were significantly more likely to have undetectable HBV DNA compared to HDAb-negative patients. This phenomenon, known as “viral dominance” or suppression, occurs because the HDV large antigen (L-HDAg) and p24 proteins repress the transcriptional enhancer of HBV, thereby inhibiting HBV replication [20]. Similar patterns of low HBV DNA despite advanced liver disease have been reported in HDV-endemic African cohorts, underscoring the limitations of HBV DNA-based treatment algorithms in such settings [8]. This has critical clinical implications: relying solely on HBV DNA levels to guide treatment decisions in Mauritania is insufficient. Clinicians may underestimate the severity of liver disease in patients with low HBV viral loads if HDV status is unknown, leading to missed opportunities for intervention in those at highest risk of progression.

The clinical impact of this co-infection is evident in the disproportionate burden of advanced liver disease among HDV-positive individuals. In our cohort, HDV antibodies were present in 86.4% of cirrhotic patients and 82.4% of those with HCC. This is consistent with meta-analyses showing that HDV co-infection triples the risk of HCC and accelerates the progression to cirrhosis by approximately a decade compared to HBV mono-infection [21,22]. In line with these findings, our multivariable analysis showed that HDV antibody positivity was strongly associated with advanced liver disease, with borderline statistical significance, after adjustment for demographic, virological, and treatment-related factors. Notably, HDV seropositivity remained high even in late disease stages, while HDV RNA detectability was more variable, suggesting fluctuating replication during disease progression [8]. The aggressive nature of the disease in our study population is further highlighted by the demographic profile of HCC patients, who were on average 10 years older than cirrhotic patients, reflecting the natural history of the disease where chronic inflammation eventually culminates in malignancy.

Demographically, we observed a significant association between White Moor ethnicity and HDV positivity (approximately 80% of HDAb+ cases). This ethnic clustering has been noted previously [8] and warrants further anthropological and genetic investigation. It may reflect specific cultural practices facilitating horizontal transmission in early childhood or founder effects within this community. Additionally, the male predominance in advanced disease stages (cirrhosis and HCC) observed here is a well-documented phenomenon in chronic viral hepatitis, attributed to hormonal factors and higher rates of synergistic risk factors such as smoking or metabolic comorbidities in men [23].

Despite the severity of disease presentation—with over a quarter of the cohort already cirrhotic—access to antiviral therapy remains suboptimal. Nearly 70% of our patients were treatment-naïve at presentation. While tenofovir (TDF) is effective at suppressing HBV DNA, it has limited efficacy against HDV itself [24]. The current standard of care available in many low-resource settings, PEG-IFN, offers poor cure rates for HDV. However, the emergence of novel entry inhibitors like bulevirtide offers hope, though cost and availability remain significant barriers in sub-Saharan Africa [25]. Taken together, our findings underscore the urgent need for Mauritania to prioritize systematic HDV screening among all HBsAg-positive individuals, regardless of HBV viral load, alongside earlier diagnosis and improved access to effective current and emerging therapies, in order to reduce the substantial burden of cirrhosis and hepatocellular carcinoma [5,26].

The lower burden of HBV and HDV infection observed among younger individuals may partly reflect the successful implementation of hepatitis B vaccination in Mauritania, which has achieved high pediatric coverage in recent years. National data indicate a substantial decline in HBsAg prevalence among vaccinated children, supporting the effectiveness of early-life immunization. As HDV transmission depends strictly on HBV infection, sustained HBV vaccination coverage is expected to contribute to a long-term decrease in HDV prevalence and associated liver disease [27].

### Limitation

This study has limitations inherent to its design. As a hospital-based study conducted at a tertiary referral center (INHV), referral-based selection bias toward symptomatic or advanced cases, as well as toward patients with the most complete data, is likely, potentially leading to an overestimation of cirrhosis and hepatocellular carcinoma (HCC) prevalence compared with the general population. Limited availability of hepatitis delta virus (HDV) antibody testing may have affected the accuracy of co-infection prevalence estimates. Finally, the absence of HBV and HDV genotype data precluded assessment of the impact of viral strains on disease progression.

## 5. Conclusions

This study demonstrates that Mauritania is a hyper-endemic setting for hepatitis delta virus, with nearly one-third of HBsAg-positive patients harboring HDV infection. HDV co-infection is strongly associated with advanced liver disease and accounts for the majority of cirrhosis and hepatocellular carcinoma cases, despite frequent suppression of HBV replication. Patients exhibit a broad clinical spectrum, yet often present late, with a high proportion already cirrhotic or decompensated and largely untreated at diagnosis. These findings highlight the limitations of HBV DNA-based disease assessment and underscore the urgent need for systematic HDV screening, earlier diagnosis, and improved access to effective therapies to reduce the burden of advanced liver disease in Mauritania.

## Figures and Tables

**Table 1:** Socio-demographic characteristics of study participants.

Characteristics	Category	Total Cohort
Population size (*n*)		401
Age (years) (*n*, %) (M ± SD)	All Samples 17 ≤ AGE ≤ 25 (*n* = 42, 10.47%) 25 < AGE ≤ 40 (*n* = 176, 43.9%) 40 < AGE ≤ 65 (*n* = 172, 42.9%) AGE > 65 (*n* = 11, 2.7%)	40.479 ± 12.145 22.83 ± 1.99 33.38 ± 4.285 50.09 ± 6.396 71 ± 7.183
Gender *n*, (%)	Male Female	203 (50.62%) 198 (49.38%)
Ethnic group *n*, (%)	White Moor Black Moor	278 (69.33%) 123 (30.67%)
Lifestyle *n*, (%)	Urban Rural	153 (38.16%) 129 (32.17%)
Marital status *n*, (%)	Single Married	79 (19.7%) 310 (77.31%)
Educational attainment *n*, (%)	Formal/traditional education No formal/traditional education	207 (51.6%) 167 (41.7%)
Occupation’s category *n*, (%)	Public service Trade/private sector Jobless	40 (9.98%) 96 (23.9%) 39 (9.73%)

M: Mean; SD: Standard Deviation; *n*: number of patients with available data; Educational attainment: attendance at any level of primary, traditional (Mahdara), secondary, high school, or university education; no formal or traditional education: no attendance at any formal or traditional schooling.

**Table 2:** Clinical spectrum.

Clinical Stage	*n*	% (95% CI)
All	401	
CHB	265	66.1% (61.2–70.7)
Cirrhosis	110	27.4% (23.1–32.1)
HCC	17	4.2% (2.5–6.7)

CHB: Chronic hepatitis B; HCC: Hepatocellular carcinoma; 95% CI: Confidence Interval of 95%.

**Table 3:** Clinical and virological characteristics of study cohort.

Characteristics	Category	Total Cohort
Clinical features *n*, (%)	Decompensation symptoms Jaundice Asymptomatic	80 (19.95%) 46 (11.47%) 275 (68.58%)
Ultrasound	Abnormal ultrasound Abnormal ultrasound; Signs of portal hypertension Normal ultrasound	69 (17.21%) 104 (25.94%) 228 (56.86)
Blood markers *n*; Me, (IQR)	AST (IU/L) ALT (IU/L) Bilirubin (mg/L) PT (%) γGT (IU/L) AFP (ng/mL) Urea (g/L) Creatinine (mg/L)	401; 34 (66) 401; 38 (53.8) 316; 12.05 (12.05) 341; 86 (22) 313; 62 (60.52) 307; 10.1 (18.45) 327; 0.32 (0.195) 327; 11 (4.7)
HDAb Status *n*; (%)	HDAb Positive HDAb Negative ND	128 (31.92%) 111 (27.68%) 162 (40.4%)
HDV RNA *n*; Me (IU/mL), (IQR), (%)	HDV RNA Positive HDV RNA Negative (*n*, %) ND	65; 270 (140,390) (50.8%) 63 (49.2%) 273 (68.08%)
HBV DNA *n*; Me (IU/mL) (IQR); (%)	HBV DNA Detectable HBV DNA > 2000 IU/mL HBV DNA Undetectable	233; 876 (4372.5); 58.105% 90; 5723.25 (42,739.25); 22.4% 168; 41.895%
Treatment *n*; (%)	PEG-INFα PEG-INFα + TDF TDF Naive	101 (25.187%) 13 (3.242%) 8 (1.99%) 279 (69.576%)

Me: Median; IQR: Interquartile; *n*: number of patients with available data. AFP: Alpha-fetoprotein; γGT: Gamma-glutamyl transferase; AST: Aspartate aminotransferase; ALT: Alanine aminotransferase; HDAb: Hepatitis Delta Antibody; PEG-INFα: α-Pegylated Interferon-alpha; TDF: Tenofovir disoproxil fumarate; PT: Prothrombin Time (expressed as the percentage of normal prothrombin activity). (%): proportion among tested individuals. IU: International Unit. ND: Not determined.

**Table 4:** Characteristics of patients in this study according to HDAb status.

Characteristics	Categories	HDAb-Positive	HDVAb-Negative	*p*-Value ^a^
*n*	239	128	111	-
Age (years) (M ± SD)		43.461 ± 11.4	40.135 ± 11.45	0.025
Gender (*n*)	Male Female	86 42	56 55	0.009
Ethnic Group (*n*)	White Moor Black Moor	103 25	49 62	<0.001
Marital Status (*n*)	Single Married	31 90	27 79	0.980
Clinical features (*n*)	Decompensation Signs Jaundice Asymptomatic	71 27 30	9 19 83	<0.001
Blood Markers Me, (IQR)	AST (IU/L) ALT (IU/L) γGT (IU/L) AFP (ng/mL) Total Bilirubin (mg/L) PT (%)	95 (84.25) 58.5 (46.05) 121(163.1) 39.5 (74.46) 112 (54.85) 61.21 (41.5)	25.4 (46.7) 28 (44.35) 46.6 (43) 7.25 (8.375) 9.35 (6.725) 86 (19)	<0.001 <0.001 <0.001 <0.001 <0.001 <0.001
HBV DNA (*n*)	HBV DNA Detectable HBV DNA Undetectable	50 78	96 15	<0.001
Clinical Stage	Cirrhosis/HCC CHB/Hepatitis B	109 19	18 93	<0.001
Treatment (*n*)	Under treatment Naive	107 21	15 96	<0.001

^a^ = student test or Mann–Whitney test or chi-square test according to the distribution and variables. HDAb: Hepatitis Delta Antibody; M: Mean; SD: Standard Deviation; Me: Median; IQR: Interquartile; AST: Aspartate aminotransferase; ALT: Alanine aminotransferase; γGT: Gamma-glutamyl transferase; AFP: Alpha-fetoprotein; PT: Prothrombin Time; HCC: Hepatocellular carcinoma; CHB: Chronic hepatitis B.

**Table 5:** Adjusted odds ratios (aOR) from multivariable logistic regression for factors associated with cirrhosis/HCC in HBsAg-positive patients.

Variable	aOR	95% CI	*p*
Age, per year	0.85	0.72–1.01	0.066
HDVab-positive vs. -negative	18.4	0.96–351.3	0.053
Sex *	-	-	>0.9
Ethnic group *	-	-	>0.9
HBV-DNA detectability *	-	-	>0.9
Treatment status *	-	-	>0.9

* Sex, ethnic group, HBV-DNA detectability and treatment status included as adjustment variables; estimates were unstable because of sparse cells and quasi-complete separation.

**Table 6:** Clinical and demographic characteristics of study participants suffering from cirrhosis and HCC.

Characteristics	Category	Cirrhosis	HCC
*n*		110	17
Age (years) (M ± SD)		42.673 ± 10.781	52.235 ± 8.871
Gendre *n*, (%)	Male Female	73 (66.36) 37 (33.64)	13 (76.47) 4 (23.53)
Ethnic Group *n*, (%)	White Moor Black Moor	95 (86.36) 15 (13.64)	13 (76.47) 4 (23.53)
Lifestyle *n*, (%)	Urban Rural	68 (61.8) 33 (30)	10 (58.82) 7 (41.18)
Marital Status *n*, (%)	Single Married	29 (26.36) 70 (63.64)	2 (11.77) 14 (82.35)
Clinical features *n*, (%)	Sign(s) of liver decompensation Jaundice Asymptomatic	68 (61.82) 23 (20.91) 19 (17.27)	12 (70.59) 2 (11.77) 3 (17.65)
Blood Markers Me, (IQR)	AST (IU/L) ALT (IU/L) Bilirubin (mg/L) PT (%) γGT (IU/L) AFP (ng/mL) Urea (g/L) Creatinine (mg/L)	101 (80) 64 (47) 42 (66) 49.5 (36.25) 138 (165.22) 36.2 (66.16) 0.44 (0.5) 16.6 (14.53)	113 (77) 65 (48.4) 26.22 (23.7) 76 (23) 178 (248.28) 249.7 (511.4) 0.58 (0.39) 19 (20)
HDAb Status *n*; (%)	HDAb-Positive HDAb-Negative	95 (86.36) 15 (13.64)	14 (82.35) 3 (17.65)
HDV ARN Me (IU/mL), (IQR)	HDV RNA Detectable Undetectable	10 (85,871.5) *n* = 43 (39.09%)	212,253 (6.931 × 10^+7^) *n* = 6 (35.29%)
HBV DNA Me (IU/mL), (IQR)	HBV DNA Detectable HBV DNA > 2000 IU/mL HBV DNA Undetectable	4970 (23,709) 7641 (49,282.5) *n* = 77 (70%)	1240 (2866.75) 3354 (11,259.75) *n* = 10 (58.82%)
Treatment *n*; (%)	PEG-INFα PEG-INFα + TDF TDF Naïve	85 (77.27) 13 (11.82) 7 (6.36) 5 (4.55)	16 (94.12) 0 1 (5.88) 0

M: Mean; SD: Standard Deviation; Me: Median; IQR: Interquartile; AFP: Alpha-fetoprotein; γGT: Gamma-glutamyl transferase; AST: Aspartate aminotransferase; ALT: Alanine aminotransferase; HDAb: Hepatitis Delta Antibody; HCC: Hepatocellular carcinoma; PEG-INFα: α-Pegylated Interferon-alpha; TDF: Tenofovir disoproxil fumarate; PT: Prothrombin Time.

## Data Availability

The data presented in this study are available from the corresponding author upon reasonable request, subject to ethical approval and data protection regulations.

## Abbreviations

The following abbreviations are used in this manuscript:

AFP	Alpha-fetoprotein
ALT	Alanine aminotransferase
AST	Aspartate aminotransferase
TB	Total bilirubin
γ-GT	Gamma-glutamyl transferase
PT	Prothrombin time
ELISA	Enzyme-linked immunosorbent assay
HBsAg	Hepatitis B surface antigen
HBV	Hepatitis B virus
HDV	Hepatitis delta virus
HDAb	Hepatitis delta antibody
HCC	Hepatocellular carcinoma
INHV	National Institute of Hepato-Virology (Nouakchott)
IQR	Interquartile range
qPCR	Quantitative polymerase chain reaction
RT-qPCR	Reverse-transcription quantitative PCR
S-HDAg	Small hepatitis delta antigen
SD	Standard deviation
TDF	Tenofovir disoproxil fumarate
VL	Viral load
WHO	World Health Organization
